# Vanadium for Green Energy: Increasing Demand but With Health Implications in Volcanic Terrains

**DOI:** 10.1029/2021GH000579

**Published:** 2022-07-01

**Authors:** John Parnell

**Affiliations:** ^1^ School of Geosciences University of Aberdeen Aberdeen UK

**Keywords:** vanadium, groundwater, volcanic rocks, Argentina

## Abstract

The transition to a clean energy future may require a very substantial increase in resources of vanadium. This trend brings into focus the potential health issues related to vanadium in the environment. Most vanadium enters the Earth's crust through volcanic rocks; hence, vanadium levels in groundwaters in volcanic aquifers are higher than in other aquifers and can exceed local guidance limits. The biggest accumulation of volcanogenic sediment on the planet is downwind of the Andes and makes up much of Argentina. Consequently, groundwaters in Argentina have the highest vanadium contents and constitute a global vanadium anomaly. The high vanadium contents have given rise to health concerns. Vanadium could be extracted during remediation of domestic and other groundwater, and although the resultant resource is limited, it would be gained using low‐energy technology.

## Key Points

- Groundwaters in volcanic terrains contain high vanadium contents
- Vanadium contents are acutely high in Argentina due to inheritance from volcanic rocks in the Andes
- Increased demand for vanadium in the green energy revolution highlights the need for awareness of potential contamination of groundwater

## 1 Introduction

The high demand for vanadium in support of the green energy revolution will require a substantial increase in mining for new resources, possibly a 200% rise in annual demand by 2050 (World Bank, 2020). Vanadium is needed for vanadium‐flow batteries, potentially on a very large scale (Colthorpe, 2021; Gencten & Sahin, 2020; Zhang et al., 2019), in addition to the established demands for high‐strength steel and electronics. However, there is an increasing awareness of the possible health hazards of high levels of vanadium in the environment (Amuah et al., 2021; Mitchell et al., 2011; Vasseghian et al., 2021; Ścibior et al., 2021). Excessive amounts of vanadium in the human body can affect the digestive system, the urinary tract, and the reproductive system and for example, cause anemia, kidney disease, asthma, dermatitis, and rhinitis (Jayawardana et al., 2015; Wilk et al., 2017; Yang et al., 2017). Exposure to vanadium has also been implicated in birth defects (Hu et al., 2017). Health problems due to vanadium in groundwater may be exacerbated by co‐occurrence with other toxic elements (Chen et al., 2022; Coyte & Vengosh, 2020). The concerns will rise as more vanadium mining takes place. The bulk of vanadium is obtained from ores in volcanic rocks containing the mineral titanomagnetite, in which vanadium is a trace element (Gilligan & Nikoloski, 2020; Yang et al., 2021). There are specific concerns about the consequences for human health of mining and processing titanomagnetite ores for vanadium (Makhotkina & Shubina, 2017; Yang et al., 2014; Yu & Yang, 2019; Zhang et al., 2020). There is an appreciation that volcanic activity in general introduces trace elements, including vanadium, that can be toxic to humans (Duntas, 2016; Nahar, 2017).

The aims of this study are to:

1. Review data about V contents of groundwater in volcanic rocks in comparison with other nonvolcanic groundwaters and legislative limits for drinking water.
2. Assess where the occurrence of V‐rich groundwaters in volcanic rocks might be most acute.
3. Make a preliminary assessment of whether the cleanup of high V contents in groundwaters could be linked to the extraction of V as a resource.

## 2 Groundwater in Volcanic Rocks

**Figure 1:**
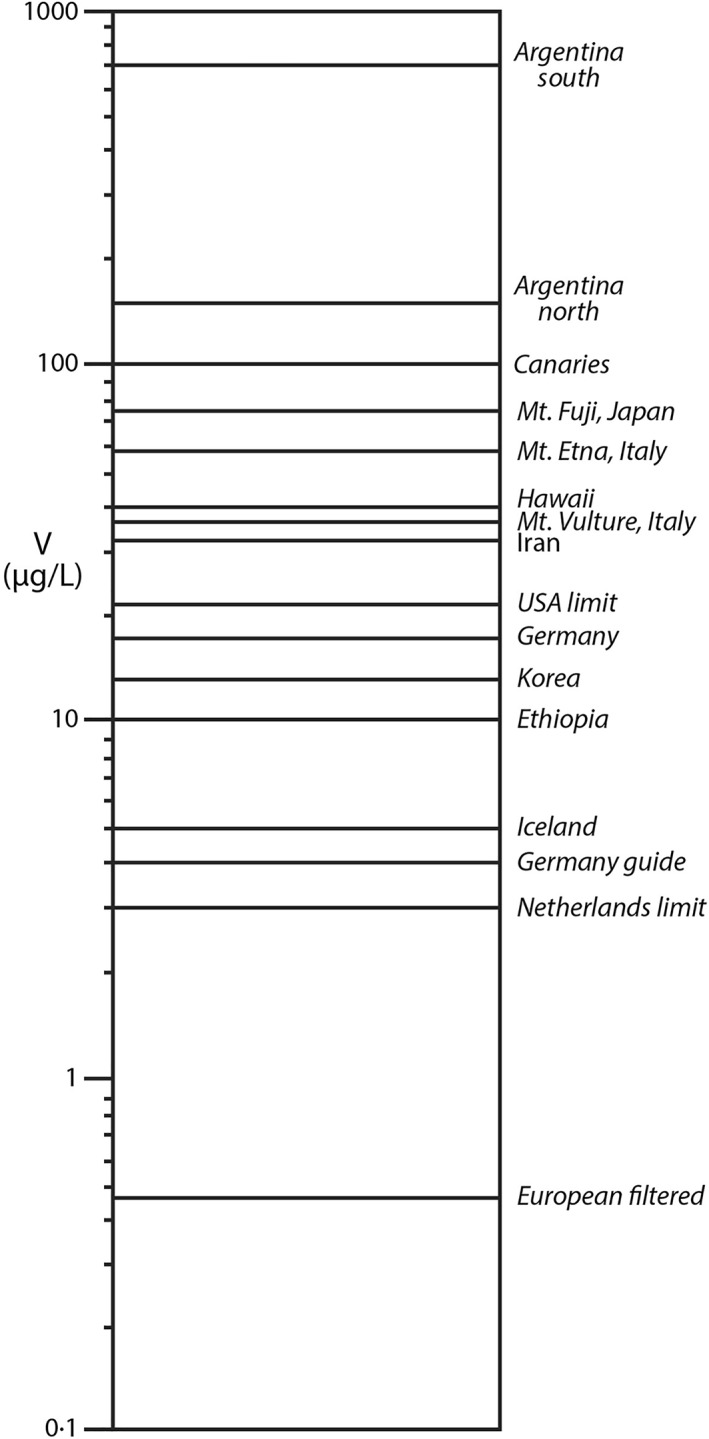
Vanadium contents (μg/L) in groundwater in aquifers in volcanogenic rocks compared to groundwaters in nonvolcanic aquifers and national legal/guidance limits for drinking water. Note that the scale is logarithmic.

The high input of volcanic matter is potentially an environmental problem for Argentina. In other parts of the world, where groundwater occurs in volcanic rocks, the water contains anomalously high amounts of vanadium (Table 1; Figure 1). The water becomes enriched in vanadium by dissolution of the volcanic minerals and glass. The benchmarks against which to assess water compositions vary between countries (Table 2) but values include a health reference limit of 21 μg/L in tap water by the U.S. Environmental Protection Agency (Environmental Working Group, 2021) and lower limits for groundwater in some European countries (Länderarbeitsgemeinschaft Wasser, 2004; Smit, 2012). There is no statutory limit for the whole European Union, where vanadium values in some Italian groundwaters would probably exceed any limit due to volcanic activity (Crebelli & Leopardi, 2012). In Britain and Europe, mean groundwater vanadium contents are less than 1 μg/L (MacDonald et al., 2017; Shand et al., 2007; Smit, 2012). A content of >15 μg/L vanadium in drinking water has been suggested as a potential health risk in the State of California, USA (Gerke et al., 2010).

**Table 1:** Mean Groundwater Contents of Vanadium in Aquifers in Volcanic Rocks and Detailed Data for Groundwater in Argentina and Adjacent Regions

Country/Province	Volcanics	Data source	V (μg/L)	Data points	Reference
Italy, Central	Plio‐Pleistocene	Water in volcanics	13	214	Cinti et al., 2015
Italy, Central	Plio‐Pleistocene	Water in volcanics	36	7	Sappa et al., 2014
Italy, Mt. Etna	Recent	Water in volcanics	57	10	Marczewski et al., 2015
Italy, Mt. Vulture	Pleistocene	Water in basalt, pyroclastics	36	34	Parisi et al., 2011
Serbia	Paleogene	Water in andesites	7.9	2	Petrovic Pantić et al., 2015
Iran	Quaternary	Water in andesites	32	16	Ghoreyshinia et al., 2020
Ethiopia	Quaternary	Hot springs in volcanics	10	12	Rango et al., 2010
Djibouti	Quaternary	Water in basalts	65	13	Ahmed et al., 2017
Tanzania	Recent	Water in volcanics	18	48	Tomašek et al., 2022
Canary Islands, El Hierro	Quaternary‐Recent	Water in basalts	101	173	Luengo‐Oroz et al., 2014
Madeira, Porto Santo	Miocene	Water in basalts and hyaloclastites	109	16	Condesso de Melo et al., 2020
Iceland	Recent	Water in volcanics	5	166	Barbieri et al., 2021
Iceland, Hekla	Recent	Water in volcanics	16	4	Holm et al., 2010
Korea, Jeju	Quaternary	Water in volcanics	13	53	Koh et al., 2016
Japan, Mt. Fuji	Pleistocene‐Recent	Water in volcanics	64	5	Kato et al., 2004
Kamchatka, Russian Far East	Recent	Water in thermal springs	219	6	Bortnikova et al., 2009
Hawaii	Holocene	Water in lava tubes	74	5	Prouty et al., 2017
Hawaii	Holocene	Groundwater	40	12	McCleskey et al., 2020
USA, northwestern	Miocene (Columbia River)	Water in basalts	∼10	>20	Newcomb, 1972
Scotland, UK	Devonian, Carb., and Paleogene	Water in volcanics	2.2	29	MacDonald et al., 2017
Germany, Eifel	Pleistocene	Water in volcanics	17	7	Härter et al., 2020
Germany, Saar‐Nahe Basin	Permo‐Carboniferous	Water in mixed volcanics and sediment	19–48	range	Leiviskä, 2021
Argentina (Tucumán)		Shallow wells Aquifers	31–300 (median 77)	42	Nicolli et al., 2012
			45–162 (median 64)	17	
Argentina (Salta)		Aquifer spring	1–15 (median 4 and **mean 6**)	10	Concha et al., 2010
Argentina (Córdoba)		Aquifers Aquifers	10–670 (median 30 and **mean 66**)	66	Farías et al., 2003
			30–2,710 (**mean 995**)	9	Pérez‐Carrera & Cirelli, 2013
Argentina (Santiago del Estero)		Aquifers	6–1,003 (median 35 and **mean 132**)	37	Bhattacharya et al., 2006
Argentina (Santa Fe)		Aquifers	76–1,090 (median 160 and **mean 249**)	15	Siegfried et al., 2015
Argentina (Chubut)		Aquifers	100–2,500 (median 800 and **mean 918**)	14	Del Pilar Alvarez & Carol, 2019
Argentina (Buenos Aires)		Aquifers	50–2,470 (median 510 and **mean 548**)	101	Fiorentino et al., 2007
			13–1,380 (**mean 430**)	12	Bonorino et al., 2008
			40–800	10	Espósito et al., 2011
			141–556 (median 325 and **mean 330**)		Puntoriero et al., 2014
Argentina (Neuquen)		Aquifers	106–1,184 (median 146 and **mean 266**)	8	Farnfield et al., 2012
Argentina (San Luis)		Aquifers	27–164 (**mean 72**)	11	Galindo et al., 2007
Argentina (Rio Negro)		Aquifers	1–113 (median 64 and **mean 30**)	20	Al Rawahi & Ward, 2017
Argentina (La Pampa)		Aquifers	20–5,400 (median 560 and **mean 840**)	108	Smedley et al., 2002
			211–4,889 (median 1,486 and **mean 1,620**)	30	Al Rawahi & Ward, 2017
			1,156–2,472 (**mean 1,749**)	3	Jaafar et al., 2018
			20–1,972 (**mean 351**)	32	Alcaine et al., 2020
Argentina (Chaco)		Aquifers	bdl–2,646 (median 76 and **mean 204**)	45	Giménez et al., 2013
Bolivia		Aquifers	1–40 (median 8 and **mean 11**)	19	Muñoz et al., 2013
Uruguay		Aquifers, potable	3–167 (median 23 and **mean 40**)	46	Machado et al., 2020
Paraná, S. Brazil		Aquifers	5–135 (**mean 22**)	18	Rezende et al., 2019

**Table 2:** Baseline Values of Vanadium (μg/L) Contents in

Region	Baseline values	V Content (μg/L)	Reference
England/Wales	Groundwaters	Median <1	Shand et al., 2007
Scotland	Groundwaters	Median 0.4	MacDonald et al., 2017
Sweden	Bottled water	Median 0.39	Rosborg et al., 2005
Europe	Filtered water	Median 0.46	Smit, 2012
USA (Environmental Protection Agency)	Health reference concentration	21	Environmental Working Group, 2021
USA	Benchmark values	20 chronic (long‐term exposure)	Suter & Tsao, 1996
		280 acute (short‐term exposure)	
USA	Potential health risk	15	Gerke et al., 2010
Netherlands	Legal limit	3	Smit, 2012
Germany	Guide limit	4	Länderarbeitsgemeinschaft Wasser, 2004
Croatia	Drinking water limit	5	Demetriades et al., 2012
Serbia	Drinking water limit	1	Demetriades et al., 2012
China	Legal limit	50	Li et al., 2020

The collated data for mean groundwater compositions in volcanic aquifers from several parts of the world show that:

1. The vanadium values are consistently higher than in nonvolcanic aquifers as represented by the British and European values.
2. In several cases, the vanadium values exceed the statutory limits set by some countries.
3. Aquifers in old volcanic rocks from the pre‐Pleistocene geological record also show values higher than nonvolcanic aquifers.

In several of these regions, there is concern about the importance of groundwater vanadium for human health, including Italy (Arena et al., 2015), Germany (Härter et al., 2020), and the Canary Islands (Luengo‐Oroz et al., 2014).

## 3 Andean Mineralization and Groundwater in Argentina

Given the implication of greater V contents and thus greater potential implications for health, in groundwaters in volcanic rocks, we can predict where this issue might be most acute. The extent of volcanic rocks, or sediments derived from volcanic rocks, is imposed by plate tectonics and patterns of plates on Earth over the last 100 million years. The requirements for very extensive volcanic‐related aquifers are (a) long‐term plate boundary subduction, causing long‐term volcanic activity; (b) long length of boundary, as opposed to the short arcs that typify the west Pacific Rim; and (c) continued uplift to promote erosion of volcanics and their deposition as a sediment wedge, built up above sea level. These requirements are met most clearly in South America, where the Andes represent over 8,000 km subduction trench length, persistent volcanic activity, and uplift (Sundell et al., 2019) that have sourced sediment to the east. The Andes have been a plate margin mountain chain for tens of millions of years (Evenstar et al., 2015) and have shed enormous volumes of sediment eastward across Argentina. Importantly, Westerly winds have supplemented the eroded volcanic sediments with volcanic glass and ash (Mingari et al., 2017). Petrographic studies confirm that the sediment in Argentina and Chile contains volcanic debris derived from the Andes by mechanical erosion (Gómez et al., 2020; Horton, 2018) and volcanic glass. Magnetite grains in the sediment, in which much vanadium may be exported from the Andes, show evidence of alteration (Flint et al., 1986), which would have released the vanadium into groundwaters.

**Figure 2:**
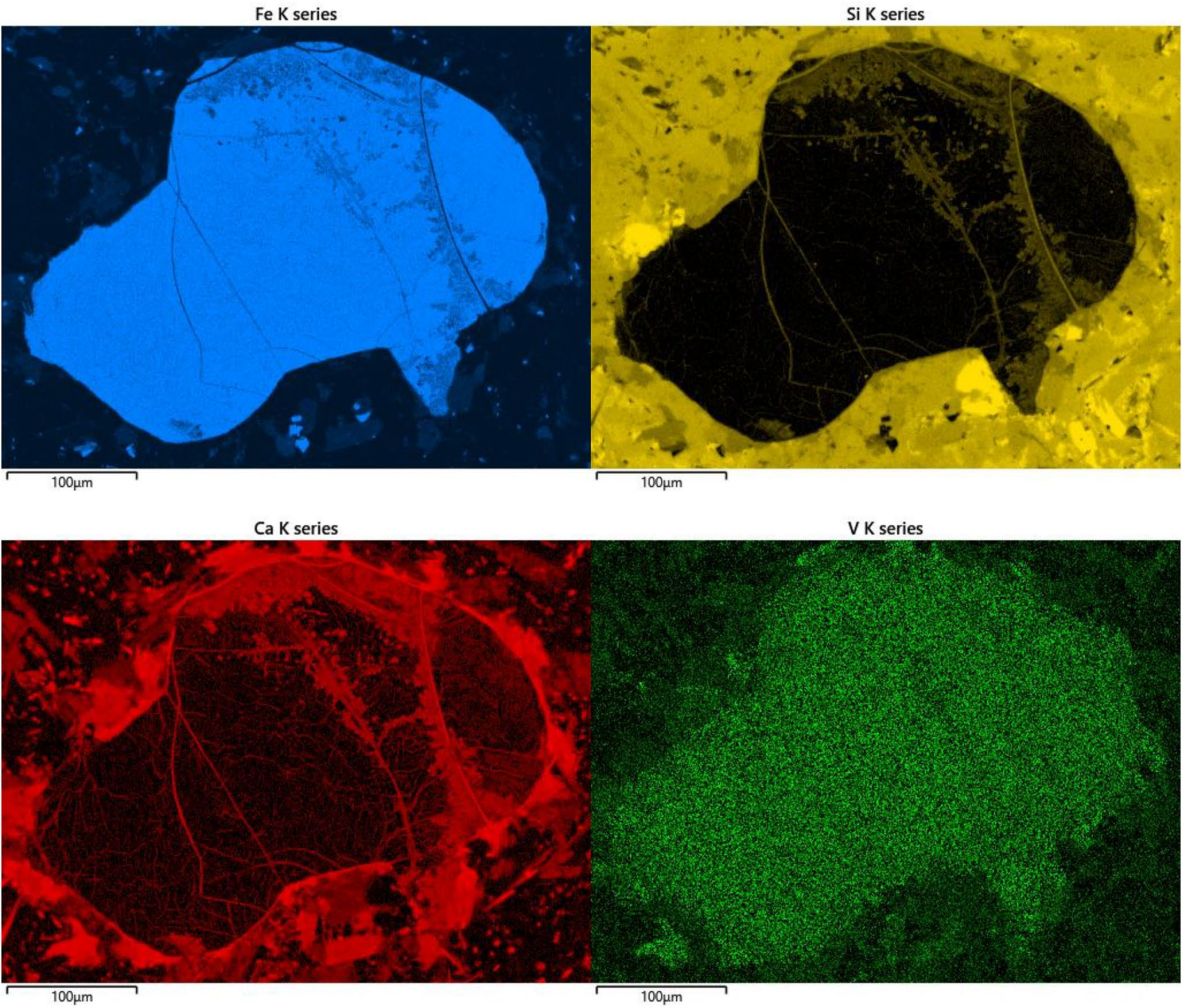
Element maps (Fe, Si, Ca, and V) for magnetite grains in andesite, Quebrada Cerrillos, Copiapo, Chile. Maps for Fe and Si show alteration, especially along fractures, and growth of Si‐rich alteration phases. Map for Ca also shows mineral alteration along fractures and additionally beyond periphery of grain. Map for V shows spread of V beyond periphery into the altered area.

Reserves of vanadium‐bearing titanomagnetite are greatest in South Africa, Russia, and China (Summerfield, 2019; Yang et al., 2021). In addition, large amounts of titanomagnetite in the Chilean Andes are mined for iron ore and contain high levels of vanadium (Broughm et al., 2017; La Cruz et al., 2020; Palma et al., 2020). Mining of iron and copper in the Andes has caused its own health concerns (Carkovic et al., 2016; Cortés et al., 2021; Reyes et al., 2020; Tapia et al., 2018). In addition to the release of toxic metals from mining spoil, the same mineralized volcanic rocks have been releasing trace elements into the environment through natural erosion over a geological timescale. The mountains are composed of magmatic rocks, which are mineralized by a range of ores including vanadium‐bearing titanomagnetite. The titanomagnetite grains exhibit alteration and dispersion of the vanadium (Figure 2). Mineral alteration in the volcanic rocks and subsequently during erosion and transport could release most or all of the vanadium into groundwaters in the sediment wedge that composes Argentina.

The influence of volcanic matter on groundwater is on a larger scale in Argentina than elsewhere. There is some local concern over vanadium contents in domestic groundwater in Argentina (Espósito et al., 2011; Jaafar et al., 2018; Nicolli et al., 2012), but this has been overshadowed by concern about arsenic contamination over much of South America (Bundschuh et al., 2021; Khan et al., 2020). However, here, we bring together diverse data sets, which show that high vanadium levels occur in groundwater across Argentina and represent the largest known region of concern for vanadium toxicity.

**Figure 3:**
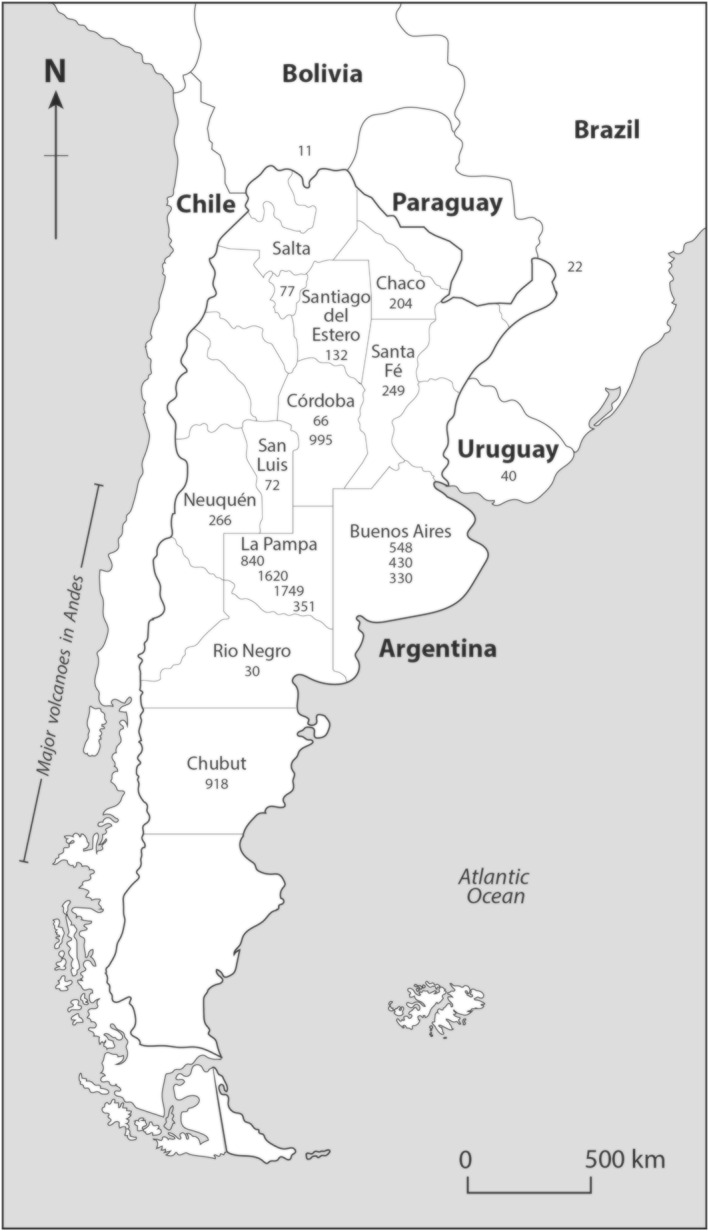
Map of southern South America, including provinces of Argentina, showing mean values for data sets of vanadium contents (μg/L) in groundwater. Data are listed in Table 1.

Twenty data sets for groundwater (Table 1) represent 12 provinces along the length of Argentina (Figure 3). The mean values for V range from 6 to 1,749 μg/L. The highest individual value is 5,400 μg V/L, recorded in La Pampa Province (Smedley et al., 2002). The lowest mean value is in Salta Province in the far north near the Bolivian border (Figure 3). There is a broad distinction between values in the northern provinces (Tucumán, Salta, Córdoba, Santiago del Estero, Chaco, Santa Fe, and San Luis) and those in the south (Buenos Aires, La Pampa, Neuquén, Rio Negro, and Chubut). The weighted mean for the northern provinces is 150 μg V/L (*n* = 235). The weighted mean for the southern provinces is 696 μg V/L (*n* = 338), nearly 5 times as high. The southern provinces lie east of the major volcanoes in the Andes (Figure 3) and they would have a greater fingerprint of their output.

The immediately surrounding countries of Bolivia, Uruguay, and southern Brazil also yield mildly anomalous groundwater data with mean values of 11, 40, and 22 μg V/L, respectively (Machado et al., 2020; Muñoz et al., 2013; Rezende et al., 2019). However, these mean values for vanadium in groundwater are lower than most of the mean values reported in Argentina and suggest a progressive decline with distance from the source of vanadium.

## 4 Mitigation

There are strategies available to mitigate high vanadium contents in groundwaters, intended primarily to cope with contamination from mining and other short‐term commercial activities. The methods include adsorption onto iron oxides, activated carbon, liquid membranes, and combinations of these materials (e.g., Kamal et al., 2017; Leiviskä, 2021; Sirviö et al., 2016; Sharififard et al., 2016). The emphasis has been in the removal of a toxic element, but there is a move toward sustainability using innovative extraction of V from wastewater and V‐rich solutions (Petranikova et al., 2020).

The mean V content of sea water is much lower than in the groundwaters discussed, commonly cited as 0.3 μg/L and up to ∼2 μg/L. The extraction of vanadium from seawater is possible (Ivanov et al., 2017; Suzuki et al., 2000) but not economically feasible. Extraction from groundwater would be more economic if the large scale was not essential. Groundwater with a V content of 100 μg V/L in 10% aquifer porosity would contain 10^4^ kg V. Given the rate at which groundwaters move in Argentina, estimated as 0.01–0.42 m/day and more specifically in one study as 0.07 m/day (Cabrera et al., 2010, 2017; Maldonado et al., 2016), groundwaters with 100 μg V/L in 10% aquifer porosity would be replenished within 40 years. Less conservative values of 20% porosity and 200 μg V/L would see replenishment of all the groundwater V within 10 years. The quantities of V that could be obtained from groundwater sources may be limited but the technology has the advantage of low temperature and low energy processing. Incidentally, the mass of V in a large ore body of 10^8^ kg vanadium in sandstone (Kelley et al., 2017) would be sourced in less than a million years in the 1 km^3^ of groundwater.

Most of the data are from groundwaters in relatively young (<5 Ma) volcanic rocks. An example that includes older (>100 Ma) volcanic rocks, in Scotland, includes groundwaters with several times the V contents for the region (MacDonald et al., 2017). However, the contents are modest compared with the values in young volcanic rocks. This implies that volcanic rocks release much of their mobile V when they are young, probably from reactive volcanic glass and unstable magmatic minerals. A further implication is that if large volumes of volcanogenic sediment can be identified in the geological record, they could have hosted V‐rich groundwaters and even sediment‐hosted V mineralization.

## 5 Conclusions

This review of V contents in groundwaters in volcanic terranes confirms previous implications that they are higher than in nonvolcanic terranes. In particular.

1. In several aquifers in volcanic rocks, the mean vanadium values exceed the statutory limits of some countries.
2. The anomalously large volume of volcanogenic sediment contributed from the long‐term erosion of the Andes is reflected in the very high groundwater V levels in Argentina.
3. The high V contents in groundwaters in young volcanic rocks suggest that the V is liberated early in the rock history.

Faced with a possibly very big increase in the demand for vanadium to support battery manufacture, new and more environmentally acceptable technologies, and new sources of vanadium, may be required. A new landscape for the processing of vanadium must take into account the potential implications for human health. The data suggest that V‐rich groundwaters may incidentally make a modest contribution to resources of the element.

## Conflict of Interest

The authors declare no conflicts of interest relevant to this study.

## Data Availability

All data are reviewed from published literature and included in this paper.
